# Theory of infectious disease spillover at an ecological boundary: impacts of seasonality and cross-boundary movement

**DOI:** 10.1098/rsos.250209

**Published:** 2025-09-17

**Authors:** Kaniz Fatema Nipa, Mozzamil Mohammed, P. Stephens, John M. Drake

**Affiliations:** ^1^ Odum School of Ecology, University of Georgia, Athens, GA, USA; ^2^ Center for the Ecology of Infectious Diseases, University of Georgia, Athens, GA, USA; ^3^ Oklahoma State University, Stillwater, OK, USA

**Keywords:** seasonality, spillover, demographic stochasticity, Ebola virus disease, human movement, continuous time Markov chain

## Abstract

Ecological boundaries are a key site for the spillover of wildlife pathogens into human and domestic livestock populations. Ebola virus is a zoonotic pathogen that is periodically introduced into humans causing outbreaks of a highly fatal haemorrhagic disease. There is evidence that spillover risk varies seasonally. Here, we hypothesize that this seasonality may be due to periodic variations in pathogen–host interactions, host social behaviours, movement and contact rates and demography. To better understand the dynamics of such a system, we studied a two-patch SIR compartmental model for the spillover of Ebola virus with seasonal and demographic variability. The model is expressed as a system of coupled ordinary differential equations (ODEs) with periodic disease transmission and dispersal between supercritical and subcritical patches. The periodic ODE system is generalized to a stochastic Continuous-Time Markov Chain (CTMC) model. The basic reproduction number of the two-patch SIR model is derived at the disease-free equilibrium to illustrate the impact of seasonality and movement on Ebola virus outbreaks. Several numerical examples are investigated. We find that the strength of seasonality and human movement are two potentially leading factors that are responsible for the intensity of periodic spillover risk from pathogen reservoirs to human settlements.

## 1. Introduction

Zoonotic diseases, infections transmitted from animals to humans, pose significant and increasing threats to public health [1–4]. These diseases, which include notable pathogens like SARS-CoV-2, avian influenza viruses and Ebola virus, often emerge unpredictably and can lead to outbreaks with severe health, economic and social consequences. Understanding the interactions between ecological, biological and social factors that drive the spillover of pathogens from animals to humans is important for predicting and mitigating future outbreaks [2].

The dynamics of zoonotic spillover, defined as the cross-species transmission of an animal pathogen into human populations, are shaped by various factors, including pathogen characteristics, host species, environmental conditions and human activities [5,6]. Wildlife reservoirs, such as bats, rodents and primates, may harbour a range of pathogens that can potentially infect humans [7]. The interface where humans interact with these wildlife hosts—through activities like hunting, agriculture and urban expansion—creates opportunities for pathogen transmission [8]. Understanding the mechanisms of spillover requires not only identifying the pathogens and their reservoirs but also examining the ecological and anthropogenic drivers that facilitate cross-species transmission.

Mathematical models have examined the movement of pathogens between different countries/zones, deriving quantities such as the basic reproduction number, stability conditions of the endemic equilibrium and conditions for the existence and persistence of the disease under different types of movement assumptions [9–16]. However, these models do not adequately represent a system where there is an endemic animal population and a human population coupled by only a small amount of contact, despite such dynamics being relatively commonplace in emerging and re-emerging infectious diseases [17,18].

A considerable body of research also acknowledges the important role played by seasonality in the transmission of infectious diseases [19–21]. It is recognized that seasonality causes fluctuations in the population dynamics, enabling similar dynamical behaviours around the same time of each year. Seasonal forces can also interact with other dynamical aspects to produce more complicated multi-annual patterns. Many infectious diseases exhibit seasonal variations in transmission due to environmental conditions such as rainfall, humidity and temperature that affect the survival of the pathogen and host susceptibility, among other factors [22–25]. Seasonal changes can also affect the movement of individuals between patches [22]. Examples of seasonally forced infectious diseases include Ebola, influenza, malaria, cholera, dengue, plague, foot-and-mouth disease, Middle East respiratory syndrome (MERS) and severe acute respiratory syndrome (SARS); see Martinez [25] for more examples.

### 1.1. Motivating example: Ebola virus disease

Ebola virus disease (EVD) is a rare and deadly disease of humans and non-human primates caused by infection with one of several viral species in the genus *Ebolavirus*, including the Ebola, Sudan, Tai Forest, and Bundibugyo viruses [26]. EVD outbreaks occur mainly on the African continent, particularly in rural areas of Central Africa [27]. Although definitive reservoirs of Ebola viruses have not been identified, contact with wildlife such as bats [28] and non-human primates [29] is a known risk factor. Once introduced into a human population, Ebola viruses can be transmitted by contact with body fluids or tissues of sick or deceased persons or animals. There are several models of Ebola transmission in human populations [24,29–42], but relatively little theory has been developed to understand the conditions that lead to spillover. Berge *et al.* [32] produced a compartmental model comprising susceptible, infected, recovered and deceased individuals which included a compartment for *Ebolavirus* in the environment. However, the application implied that the source of environmental contamination was other human cases, not an animal reservoir. Another study [40] developed a compartmental model representing individuals that are susceptible, latent, infected and that die or recover to examine the impacts of investment in medical resources on expected rates of mortality in Ebola outbreaks.

It is generally thought that Ebola spillover occurs when there is contact between a person and a nonhuman animal in the forest. For example, Stephens *et al.* [43] found that contact between humans and wild animals played a role in triggering at least 27 out of 34 (79%) Ebola virus outbreaks in human populations. In the case of Ebola, seasonality is believed to be influenced by the movement and aggregation of wildlife [22,44], with the transition between the wet and dry seasons implicated as a time of especially high spillover risk [45]. Schmidt *et al.* [45] hypothesized that seasonal pulses of fruit production attract fruit bats, primates and other susceptible species—including humans—into shared foraging areas, an idea supported by the later findings of Sundaram *et al.* [46]. These pulses act as environmental resources that concentrate both susceptible and infectious hosts, resulting in seasonal peaks in transmission. Because the same fruiting events draw multiple species, particularly humans, into the landscape, they also increase rates of movement into and between ecologically sensitive areas. Thus, seasonal movement and transmission rates are expected to be coupled, with both peaking during periods of high fruit availability and ecological interaction.

Mathematical models offer a powerful tool for dissecting the complexities of zoonotic spillover. By integrating data on pathogen biology, host ecology, and human behaviour, models can simulate various scenarios of disease emergence and spread. They provide insights into critical factors that influence spillover events, such as host density, habitat fragmentation, climate change and seasonality. Moreover, mathematical models can help identify high-risk areas and periods for zoonotic spillover, guiding surveillance and intervention strategies to prevent outbreaks before they escalate.

We present a two-patch SIR model designed to enhance our understanding of Ebola spillover risk at ecological boundaries. Our model incorporates key ecological and epidemiological parameters, allowing us to simulate the conditions under which zoonotic pathogens emerge and spread. We assumed that the population is not well mixed, but rather consists of two patches: (i) an endemic patch (i.e. a wildland patch where the virus is maintained in a nonhuman reservoir), and (ii) a human-dominated patch (i.e. settlement) where there may be human-to-human transmission, but no opportunity for contact between humans and animals. The spillover then depends on the coupling between these two patches. In mathematical models, seasonality is most naturally represented by periodically forcing the relevant dynamical coefficients, leading to time-nonhomogeneous systems of equations [47–58] which may also be stochastic [20,21,50,59–63]. Importantly, the basic reproduction number, $R_{0}$, for a nonautonomous model is typically different from that for the corresponding autonomous model. Bacaër & Ait Dads [59] show that the basic reproduction number $R_{0}$ from the underlying nonautonomous ODE model may serve as a threshold for disease extinction in the corresponding stochastic time-nonhomogeneous model. By applying our model to EVD, we aim to elucidate the role that seasonality and patch dynamics contribute to spillover, ultimately contributing to more effective prevention and control measures.

## 2. Methods

We develop and analyse deterministic and stochastic versions of a two-patch model to describe the impact of seasonal human movement and seasonal disease transmission on pathogen spillover at ecological boundaries. We consider a supercritical *reservoir* patch where the disease is endemic in animals and a subcritical human *settlement* patch connected by human dispersal. We first develop a two-patch deterministic SIR model and then investigate its utility in small populations using a continuous-time Markov chain (CTMC) model.

### 2.1. Two-patch SIR model

Following the standard formulation of the SIR model, we consider a subpopulation of size $N_{j}$ in each local patch j and divide it into three compartments: susceptible $S_{j}$, infected $I_{j}$ and recovered $R_{j}$, where j=A,B represents the reservoir and settlement patches, respectively. We assume a constant local population size in the absence of movement, with $N_{j}=S_{j}+I_{j}+R_{j}$, and constant total population size, with $N=N_{A}+N_{B}$. We further assume that all individuals are born susceptible to the disease at rate $\mu_{j}$ and die naturally (disease-independent death) at identical rate $\mu_{j}$. In numerical examples, we assume the natural birth and death rates are independent of patches (i.e. $\mu_{j}=\mu$). Following a contact between susceptible and infectious individuals, the disease is transmitted at rate $\beta_{j}$. This transmission rate is patch-specific, and its intensity depends on the region where individuals interact. The transmission rate is assumed to be high in the reservoir and relatively low in the human settlement. Infectious individuals recover at patch-independent rate γ. For simplicity, we assume that there is no disease-induced mortality since our model is focused on the investigating conditions that initially generate a spillover event.

**Figure 1.**
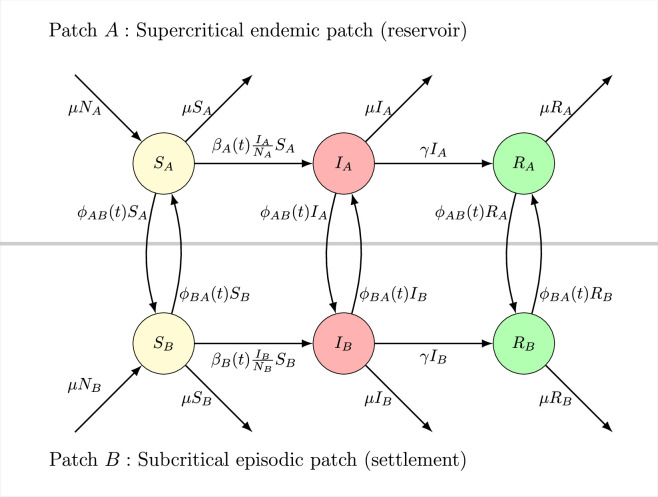
Reservoir–human interactions near an ecological boundary are described by a two-patch SIR model.

The interaction process occurs locally while individuals move across patches at dispersal rate $\phi_{jk}$, representing movement rate from patch j to patch k. All individuals (i.e*.* susceptible, infected and recovered) of the same patch type move to the other patch at an identical dispersal rate. Host species (i.e. animal reservoirs and human hosts) are not distinguished. Implicitly, we understand hosts in the reservoir patch to be from the animal reservoir (with the exception of temporary human migrants) while hosts in the settlement patch are humans (with the exception of temporary animal migrants). This model is depicted graphically in figure 1, showing possible transitions within and between patches.

### 2.2. Seasonality

Our model allows for seasonality in both disease transmission and human movement across ecological boundaries. We use the following periodic functions to describe seasonally dependent transmission and dispersal:


(2.1) $$\begin{array}{lcr} & \begin{array}{rl} \beta_{j}(t) & ={\bar{\beta }}_{j}\left(1+\sigma_{1}\cos\left(2\pi t\right)\right),\\ \phi_{jk}(t) & ={\bar{\phi }}_{jk}\left(1+\sigma_{2}\cos\left(2\pi t\right)\right),\;j,k=A,B,j\neq k, \end{array} & \end{array}$$

where $\sigma_{1}$ and $\sigma_{2}$ represent amplitudes for the transmission and the dispersal rates, respectively. We assume synchronized seasonality functions (i.e. $\sigma_{1}=\sigma_{2}=\sigma$), resulting in similar qualitative behaviour of the two functions. The baseline parameters ${\bar{\beta }}_{j}$ and ${\bar{\phi }}_{jk}$ represent the transmission and movement rates, respectively, in the absence of seasonality. The length of the seasonal period is assumed to be 1 year. Parameters of these functions are all positive.

The mathematical formulation of the two-patch deterministic model is now straightforward. It is described by six differential equations, tracing the temporal changes in the number of susceptible, infected and recovered individuals present in each local patch:


(2.2) $$\begin{array}{lcr} & \begin{array}{r} \text{Patch A}\left\{ \begin{array}{cc} \dot{S_{A}} & =\mu N_{A}-\beta_{A}(t)\frac{I_{A}}{N_{A}}S_{A}-(\mu +\phi_{AB}(t))S_{A}+\phi_{BA}(t)S_{B},\\ \dot{I_{A}} & =\beta_{A}(t)\frac{I_{A}}{N_{A}}S_{A}-(\mu +\gamma +\phi_{AB}(t))I_{A}+\phi_{BA}(t)I_{B},\\ \dot{R_{A}} & =\gamma I_{A}-(\mu +\phi_{AB}(t))R_{A}+\phi_{BA}(t)R_{B}, \end{array}\right. \end{array} & \end{array}$$

(2.3) $$\begin{array}{lcr} & \begin{array}{r} \text{Patch B}\left\{ \begin{array}{cc} \dot{S_{B}} & =\mu N_{B}-\beta_{B}(t)\frac{I_{B}}{N_{B}}S_{B}-(\mu +\phi_{BA}(t))S_{B}+\phi_{AB}(t)S_{A},\\ \dot{I_{B}} & =\beta_{B}(t)\frac{I_{B}}{N_{B}}S_{B}-(\mu +\gamma +\phi_{BA}(t))I_{B}+\phi_{AB}(t)I_{A},\\ \dot{R_{B}} & =\gamma I_{B}-(\mu +\phi_{BA}(t))R_{B}+\phi_{AB}(t)R_{A}, \end{array}\right. \end{array} & \end{array}$$

where the initial state variables are $S_{j}(0)>0$, $I_{j}(0)>0$ and $R_{j}(0)=0$, representing the number of susceptible, infected and recovered individuals at t=0, with $S_{j}(0)+I_{j}(0)+R_{j}(0)=N_{j}$. Parameters of the model are all non-negative and summarized in table 1.

**Table 1.** Numerical elasticity values describing the relative importance of each model parameter on the basic reproduction number. The basic reproduction number used in the numerical elasticity for this set of parameters is $R_{0}=4.81$.

parameters	description	values	numerical elasticity
$N_{A}$	initial population size in patch A	100	0.00
$N_{B}$	initial population size in patch B	1000	0.00
${\bar{\beta }}_{A}$	transmission rate in patch A	60	0.999
${\bar{\beta }}_{B}$	transmission rate in patch B	5	0.999
γ	recovery rate	12	−0.917
${\bar{\phi }}_{AB}$	dispersal rate of S,I,R , from patch A to B	0.4	−0.072
${\bar{\phi }}_{BA}$	dispersal rate of S,I,R , from patch B to A	0.04	−0.007
σ	seasonality amplitude	0.5	0.00
μ	natural birth and death rate	0.02	−0.004

This model is analysed both mathematically (§3) and numerically (§5) to describe the influence of the key model parameters on Ebola virus spillover risk at ecological boundaries. Particular attention is paid to exploring the influence of seasonal disease transmission and seasonal human movement on the Ebola virus spillover.

## 3. Mathematical analysis of the deterministic model

The complexity of our two-patch model precludes deriving analytical solutions. However, our analytical investigation of the disease-free equilibrium points generates insight into the long-term disease dynamics. We also calculate the basic reproduction number, $R_{0}$, using the next-generation method [64] and investigate how the model parameters influence $R_{0}$ using sensitivity analysis.

### 3.1. Disease-free equilibrium

To calculate the endemic and disease-free equilibrium points for the systems (2.2) and (2.3), we assume that all models parameters are constant. The disease-free equilibrium is typically obtained by deriving the steady-state solution of the system (2.2) and (2.3) at $I_{A}^{*}=I_{B}^{*}=R_{A}^{*}=R_{B}^{*}=0$. The solution of the resulting algebraic equations represents disease-free equilibrium point which is defined as $E_{0}=\left(S_{A}^{*},0,0,S_{B}^{*},0,0\right)$, where


(3.1) $$\begin{array}{lrr} & S_{A}^{*}=\frac{\mu^{2}N_{A}+\mu N_{A}\phi_{BA}+\mu N_{B}\phi_{BA}}{\mu^{2}+\mu \phi_{BA}+\mu \phi_{AB}}, & \\ & S_{B}^{*}=\frac{\mu^{2}N_{B}+\mu N_{A}\phi_{AB}+\mu N_{B}\phi_{AB}}{\mu^{2}+\mu \phi_{BA}+\mu \phi_{AB}}. & \end{array}$$

The initial population sizes and the dispersal rates are important determinants of the patch-dependent disease-free equilibria. In the absence of dispersal between the two patches (i.e. $\phi_{AB}=\phi_{BA}=0$), the disease-free equilibrium in each local patch is given by its initial population size (i.e. $S_{A}^{*}=N_{A}$ and $S_{B}^{*}=N_{B}$). With the onset of dispersal, the local disease-free equilibrium depends on the dispersal rates, while $S_{A}^{*}+S_{B}^{*}=N_{A}+N_{B}$, yielding the total number of individuals initially present in the system. As this holds true, it is not necessary that $S_{A}^{*}=N_{A}$ or $S_{B}^{*}=N_{B}$ with the onset of dispersal. However, the local disease-free equilibrium can be equal to the initial population size provided that $\phi AB/\phi BA=N_{B}/N_{A}$. We also calculated the epidemic equilibrium points and provide them in electronic supplementary material, appendix A.1.

### 3.2. Basic reproduction number

We use the next-generation matrix method [64] to derive an expression for the basic reproduction number, $R_{0}$ of the systems (2.2) and (2.3). The basic reproduction number is defined as the dominant eigenvalue or the spectral radius of the matrix $G=FV^{-1}$, where


$$F=\left[\frac{\partial F_{i}(E_{0})}{\partial I_{j}}\right],\text{and}\;V=\left[\frac{\partial V_{i}(E_{0})}{\partial I_{j}}\right].$$

Elements of F are new infections and elements of V are the transfer of infections among compartments [65]. All new infections and transfer rates are differentiated with respect to the state variables of the infected compartments and evaluated at the disease-free equilibria $E_{0}$ to obtain the elements of F and V.

The matrices F and V of the systems (2.2) and (2.3) are functions of time and are given by


(3.2) $$\begin{array}{lcr} & F(t)=\left(\begin{array}{cc} \beta_{A}\frac{S_{A}^{*}}{N_{A}} & 0\\ 0 & \beta_{B}\frac{S_{B}^{*}}{N_{B}} \end{array}\right) & \end{array}$$

and


(3.3) $$\begin{array}{lcr} & V(t)=\left(\begin{array}{cc} \mu +\gamma +\phi_{AB}(t) & -\phi_{BA}(t)\\ -\phi_{AB}(t) & \mu +\gamma +\phi_{BA}(t) \end{array}\right). & \end{array}$$

The next-generation matrix G is given by


(3.4) $$FV^{-1}=\frac{1}{\Delta }\left(\begin{array}{cc} \beta_{A}\frac{S_{A}^{*}}{N_{A}}(\mu +\gamma +\phi_{BA}(t)) & \beta_{A}\frac{S_{A}^{*}}{N_{A}}\phi_{BA}(t)\\ \beta_{B}\frac{S_{B}^{*}}{N_{B}}\phi_{AB}(t) & \beta_{B}\frac{S_{B}^{*}}{N_{B}}(\mu +\gamma +\phi_{AB}(t)) \end{array}\right),$$

where $\Delta =(\mu +\gamma +\phi_{AB}(t))(\mu +\gamma +\phi_{BA}(t))-\phi_{AB}(t)\phi_{BA}(t).$ Therefore, $FV^{-1}$ is non-negative and the basic reproduction number is defined as


(3.5) $$\begin{array}{lcr} & R_{0}=\rho (FV^{-1}), & \end{array}$$

where $\rho (FV^{-1})$ is the spectral radius or the dominant eigenvalue of the matrix $FV^{-1}.$ The mathematical expression for $R_{0}$ is given in electronic supplementary material, appendix A.2. See Wang & Zhao [66] for a similar derivation.

### 3.3. Sensitivity and elasticity

The basic reproduction number $R_{0}$ provides qualitative information about the initial growth of the epidemic. The sensitivity and elasticity of $R_{0}$ identify which parameters have the greatest impact on $R_{0}$ [67]. The sensitivity index of $R_{0}$ with respect to any model parameter w is defined by $\frac{\partial R_{0}}{\partial w}$. Elasticity, also known as the normalized sensitivity of $R_{0}$, measures the relative change of $R_{0}$ with respect to a parameter. Elasticity of $R_{0}$ with respect to any parameter w is defined by


$$\Upsilon_{w}^{R_{0}}=\frac{\partial R_{0}}{\partial w}\times \frac{w}{R_{0}}.$$

A positive sign for the sensitivity or elasticity index indicates that increases in the value of the parameter w will result in increases in $R_{0}$ and vice versa. A negative sign indicates that increases in the parameter value will lead to a decrease in $R_{0}$. The magnitude of the elasticity index indicates the relative importance of the parameter. These measurements are frequently used to determine the control parameters of an epidemic. More examples can be found in [67]. Elasticity values for $R_{0}$ with respect to our baseline parameter values are provided in table 1.

Based on our sensitivity analysis, we conclude that transmissibility (β) and recovery rate (γ) are typically the most important parameters affecting the dynamics of the Ebola disease dynamics. The elasticity index for the disease transmission rate and recovery rate are, respectively, 0.999 and −0.917, having the highest magnitude compared to the other parameters summarized in table 1. We note that increases in the birth, death, and dispersal rates will reduce the value of $R_{0}$ as they all have negative elasticity.

## 4. Stochastic model

Demographic stochasticity cannot be neglected in disease systems with a small number of infected individuals, such as when primary cases arise through spillover. Here, we derive a stochastic version of the deterministic two-patch model. We develop and analyse an event-driven continuous-time Markov chain model. In the model presented here, the state variables $S_{j}(t)$, $I_{j}(t)$ and $R_{j}(t)$ are discrete random variables. Birth, death and dispersal are random events that occur with probabilities determined by the rates specified in the deterministic model (summarized in table 2). The primary feature of Markov models is that the future state of the system depends only on its current state. For instance, the number of infected individuals at time (t+1) depends only on the number at the preceding time t.

**Table 2.** State transitions for each event type i and the associated transition probabilities. The transition vector is ordered as $\left(S_{A},I_{A},R_{A},S_{B},I_{B},R_{B}\right)$ .

event i	description	transition	transition probability $P_{i}$
1	infection in patch A	(−1,1,0,0,0,0)	$\beta_{A}(t)\frac{I_{A}(t)}{N_{A}}S_{A}(t)\Delta t+o(\Delta t)$
2	natural death of infected in patch A	(0,−1,0,0,0,0)	$\mu I_{A}(t)\Delta t+o(\Delta t)$
3	recovery in patch A	(0,−1,1,0,0,0)	$\gamma I_{A}(t)\Delta t+o(\Delta t)$
4	infected dispersal from patch A to B	(0,−1,0,0,1,0)	$\phi_{AB}(t)I_{A}(t)\Delta t+o(\Delta t)$
5	infected dispersal from patch B to A	(0,1,0,0,−1,0)	$\phi_{BA}(t)I_{B}(t)\Delta t+o(\Delta t)$
6	infection in patch B	(0,0,0,−1,1,0)	$\beta_{B}(t)\frac{I_{B}(t)}{N_{B}}S_{B}(t)\Delta t+o(\Delta t)$
7	natural death of infected in patch B	(0,0,0,0,−1,0)	$\mu I_{B}(t)\Delta t+o(\Delta t)$
8	recovery in patch B	(0,0,0,0,−1,1)	$\gamma I_{B}(t)\Delta t+o(\Delta t)$
9	newborn individuals in patch A	(1,0,0,0,0,0)	$\mu N_{A}\Delta t+o(\Delta t)$
10	natural death of susceptible in patch A	(−1,0,0,0,0,0)	$\mu S_{A}(t)\Delta t+o(\Delta t)$
11	susceptible dispersal from patch A to B	(−1,0,0,1,0,0)	$\phi_{AB}(t)S_{A}(t)\Delta t+o(\Delta t)$
12	susceptible dispersal from patch B to A	(1,0,0,−1,0,0)	$\phi_{BA}(t)S_{B}(t)\Delta t+o(\Delta t)$
13	newborn individuals in patch B	(0,0,0,1,0,0)	$\mu N_{B}\Delta t+o(\Delta t)$
14	natural death of susceptible in patch B	(0,0,0,−1,0,0)	$\mu S_{B}(t)\Delta t+o(\Delta t)$
15	natural death of recovered in patch A	(0,0,−1,0,0,0)	$\mu R_{A}(t)\Delta t+o(\Delta t)$
16	recovered dispersal from patch A to B	(0,0,−1,0,0,1)	$\phi_{AB}(t)R_{A}(t)\Delta t+o(\Delta t)$
17	recovered dispersal from patch B to A	(0,0,1,0,0,−1)	$\phi_{BA}(t)R_{B}(t)\Delta t+o(\Delta t)$
18	natural death of recovered in patch B	(0,0,0,0,0,−1)	$\mu R_{B}(t)\Delta t+o(\Delta t)$
19	no change	(0,0,0,0,0,0)	$1-\Sigma_{i=1}^{14}P_{i}\Delta t+o(\Delta t)$

In Markov models, the time interval between two successive events needs to be sufficiently small to ensure that at most one event occurs during this time interval [68]. Given a transmission event in patch A in a small time interval Δt, the transition probability is defined by


$$P_{1}(S_{A}(t+\Delta t)-S_{A}(t)=-1\text{ and }I_{A}(t+\Delta t)-I_{A}(t)=1)=\beta_{A}(t)\frac{I_{A}(t)}{N_{A}}S_{A}(t)\Delta t+o(\Delta t),$$

and the corresponding transition will thus be (−1,1,0,0,0,0). The same procedure applies to events (2)−(19) provided in table 2. We used the Gillespie stochastic simulation algorithm [69] to implement our continuous-time Markov chain model.

## 5. Numerical analysis

All numerical analysis was performed using the R programming language. We used the R package deSolve [70] to study the deterministic model and our own implementation of Gillespie’s direct method [69] to generate sample paths from the stochastic model. The initial population sizes were fixed for all analyses, with a small number of individuals in the reservoir patch and relatively large number of individuals in the settlement patch. In the stochastic model, fluctuations in population sizes near the extinction boundary can lead to consistent extinction of the disease. To avoid fluctuation-driven extinctions in our stochastic model, we added a positive constant to the seasonal transmission rate to ensure that the epidemic can recover with some probability when reaching its absorbing state by fluctuation near the extinction line (time axis).

### 5.1. Parameters

Notional parameter values used to study the behaviour of this model are provided in table 1. We emphasize that our model was parameterized to illustrate the key dynamical properties of the model and is not intended as a representation of any particular system. That said, it is notionally parameterized around Ebola spillover from wild animals to adjacent human settlements. Chimpanzee (*Pan troglodytes*) and Gorilla (*Gorilla gorilla*) may range from less than one individual per square kilometres to about 5 km${,}^{-1}$ [71–74]. In contrast, humans in dispersed settlements may occur in densities of 50 km${,}^{-1}$. Therefore, we suppose the population of the settlement patch to be an order of magnitude greater than the reservoir patch. For numerical simulations, we set the initial population size in patch A to be $N_{A}=100$ and in patch B to be $N_{B}=1000$. We then allowed seasonal migration between patches, taking into account that the movement rate from the settlement (patch B) to the reservoir (patch A) is relatively low. More specifically, we initially chose the baseline movement rates as ${\bar{\phi }}_{AB}=0.4$. Since the local disease-free equilibrium remains equal to the initial population size only if $\phi_{AB}/\phi_{BA}=N_{B}/N_{A}$ we therefore set ${\bar{\phi }}_{BA}=0.04$. The length of the seasonal period was fixed at 1 year, and we systematically varied the amplitude of seasonality to investigate its impact on the system dynamics.

Ebola virus has an incubation period of 1−21 days and duration of illness of approximately two weeks before recovery or death [75]. We therefore assumed a recovery rate (γ) of 12 infectious periods per year. Transmissibility (β) is often the most difficult parameter of compartmental models to estimate because it cannot be observed directly. It is often inferred through model fitting or by estimating basic or effective reproduction numbers ($R_{0}$ and $R_{e}$) and then rearranging to express β in terms of $R_{0}$ or $R_{e}$ and γ. Our expression for $R_{0}$ is too complicated to admit any such simple expression for β. Simpler models of Ebola virus outbreaks in human populations comparable to those envisioned here estimate $R_{0}$ to be typically between 1.4 and 3.7 [35]; $R_{e}$ may be virtually anything less than $R_{0}$ depending on the effectiveness of interventions. We are unaware of any estimates of $R_{0}$ for Ebola in non-human animals. Our model considers the two populations to be coupled. We therefore require two values of β: ${\bar{\beta }}_{A}$ sufficiently large that the reservoir patch is supercritical and ${\bar{\beta }}_{B}<{\bar{\beta }}_{A}$ such that the episodic patch is subcritical (as any human population with interventions to present transmission must be). For illustration, the baseline transmission rates in patches A and B were chosen as ${\bar{\beta }}_{A}=60$ and ${\bar{\beta }}_{B}=5$ yielding $R_{0}=4.81$.

## 6. Results

**Figure 2.**
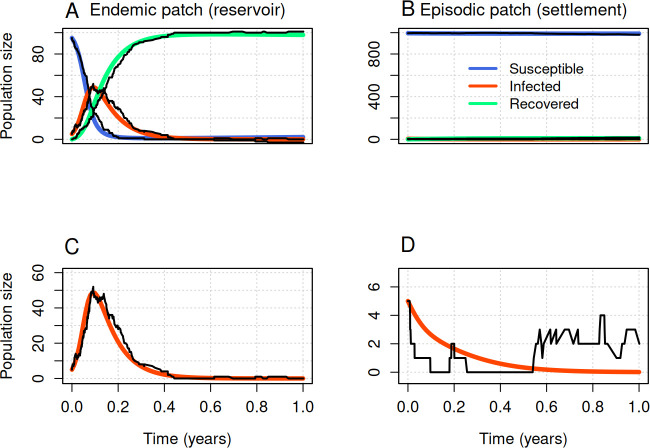
The disease goes extinct in the absence of seasonality and human dispersal. Dynamics of the deterministic model (colours) and one instance of the stochastic model (black) for a period of 1 year, without seasonality and without movement in the reservoir patch (A and C) and the settlement patch (B and D). Panels C and D show the dynamics of infectious individuals that are shown in A and B but made more visible with a truncated *y*-axis. In all plots, the *x*-axis shows time and the *y*-axis corresponds to the population size in each compartment. Initial conditions for patch A were $S_{A}=95$, $I_{A}=5$ and $R_{A}=0$, and for patch B were $S_{B}=995$, $I_{B}=5$ and $R_{B}=0$. Other parameters were μ=0.2, γ=12, $\beta_{A}=60$, $\beta_{B}=5$ and $\phi_{AB}=\phi_{BA}=\sigma =0$ (no movement, no seasonality).

We numerically analysed a two-patch model to explore disease spillover at ecological boundaries. In particular, we investigated the ecologically important roles played by seasonality in transmission and human movement. Throughout, we considered a scenario where the disease transmission rate is high in one patch (reservoir patch) and low in another patch (settlement patch) to elucidate how seasonal variations and human use of natural environments might facilitate spillover events. We initially chose parameter values in a way that the outbreak will not take off in the human settlement region in the absence of dispersal, i.e., a source–sink system (figure 2) [76]. The dispersal rate from the settlement to the reservoir patch was initially assumed to be smaller than that from the reservoir to settlement patch. The initial population size in the human settlement region was greater than that in the reservoir patch by a factor of 10, i.e. $N_{B}/N_{A}=10$. Our analytical study demonstrated that the disease-free equilibrium in each patch will be equal to the initial population size if $N_{B}/N_{A}=\phi_{AB}/\phi_{BA}$. However, we also compared these deterministic model results with a continuous-time Markov chain model.

### 6.1. Movement

**Figure 3.**
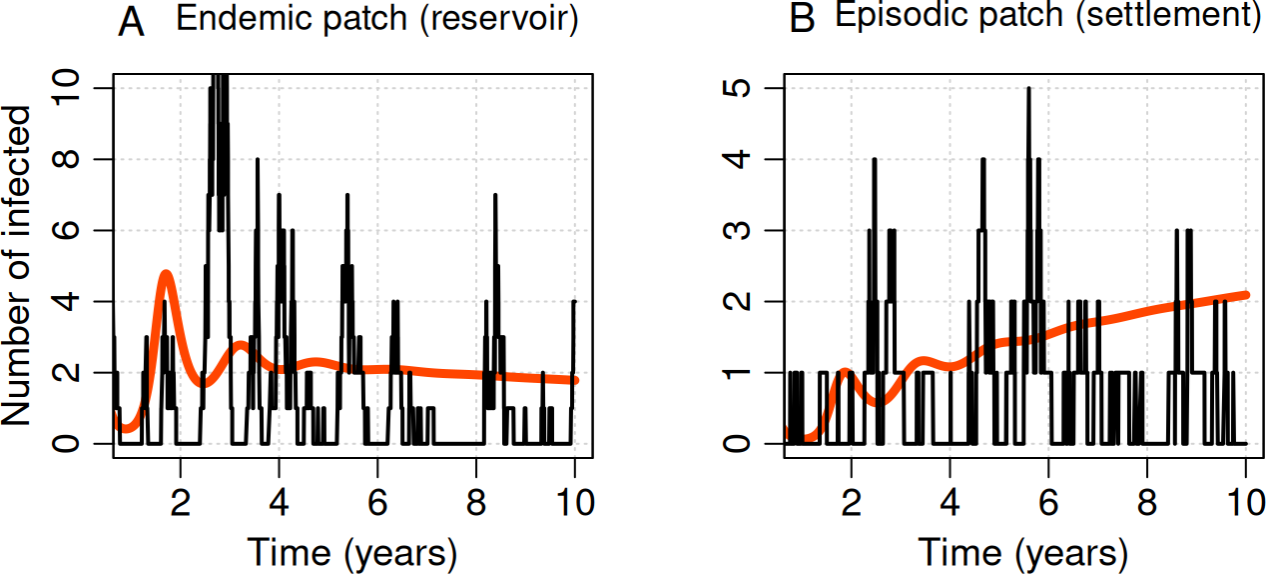
Human movement causes sporadic spillover in the subcritical region. Dynamics of the deterministic (red) and the stochastic (black) models for a period of 10 years with movement between patches, but without seasonality in either disease transmission or movement. Initial conditions for patch A were $S_{A}=95$, $I_{A}=5$, and $R_{A}=0$, and for patch B were $S_{B}=995$, $I_{B}=5$ and $R_{B}=0$. Other parameters were μ=0.2, γ=12, $\beta_{A}=60$, $\beta_{B}=5$, $\phi_{AB}=0.4$, $\phi_{BA}=0.04$ and σ=0.

Figure 2 shows the trajectory of an outbreak that represents a sample path for 1 year of the stochastic model superimposed on the trajectory of the corresponding deterministic model without human movement between the two patches and without seasonality in the disease transmission rate. Both patches were initialized with five infectious individuals. The choice of parameters for the spatially decoupled model (supercritical in the reservoir patch and subcritical in the settlement patch) unsurprisingly led to an acute outbreak in the reservoir patch leading to endemic transmission at a low level and decline in the number of infected individuals in the settlement patch leading to extinction. In the presence of human movement between the two patches, pathogens are transported from the reservoir to the settlement region, thereby allowing spillover to occur (figure 3). The model dynamics for this particular movement scenario are provided as electronic supplementary material, appendix, figure A.1, showing changes in the number of susceptible, infected and recovered, all together.

### 6.2. Seasonality

**Figure 4.**
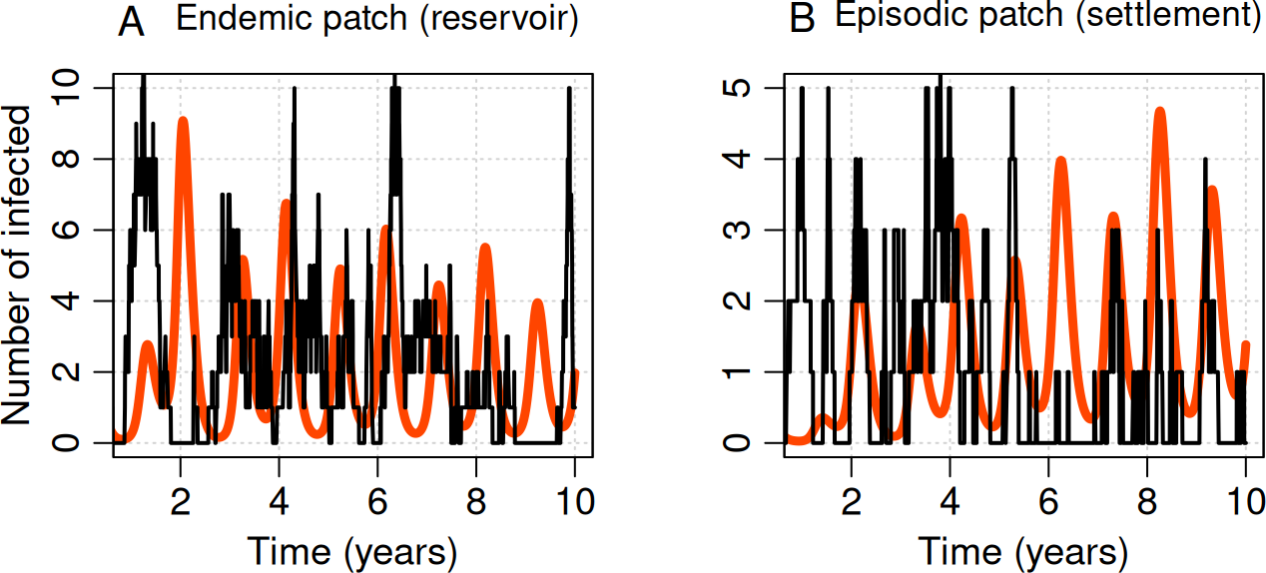
Seasonal transmission and human movement result in disease persistence. Dynamics of the deterministic (red) and the stochastic (black) models for a period of 10 years, with seasonal disease transmission and seasonal movement. Initial conditions for patch A were $S_{A}=95$, $I_{A}=5$ and $R_{A}=0$, and for patch B were $S_{B}=995$, $I_{B}=5$ and $R_{B}=0$. Other parameters were μ=0.2, γ=12, $\beta_{A}=60$, $\beta_{B}=5$, $\phi_{AB}=0.4$, $\phi_{BA}=0.04$ and σ=0.5.

With seasonal human dispersal between the reservoir and human settlement regions, the pathogen is frequently transported from the reservoir to the settlement patch, where they may result in small clusters of disease (figure 4). At the chosen parameters, these clusters invariably die out so that the disease is not maintained endemically. By enabling seasonal migration and seasonal transmission of the disease, we showed that once spillover occurs, the disease recurs even in the settled patch (figure 4B). We ran the simulation for 10 years, demonstrating that the epidemic dynamics exhibit seasonal fluctuations (figure 4). Note that, in stochastic models, how the probability of a disease extinction increases with time depends on the initial population size. In the presence of seasonal forcing, the small number of infected individuals frequently goes extinct but is subsequently reignited by additional spillovers (figure 4B). The model dynamics for this particular movement-seasonality scenario are provided as electronic supplementary material, appendix, figure A.2, showing changes in the number of susceptible, infected and recovered, all together.

### 6.3. Magnitude of seasonality

**Figure 5.**
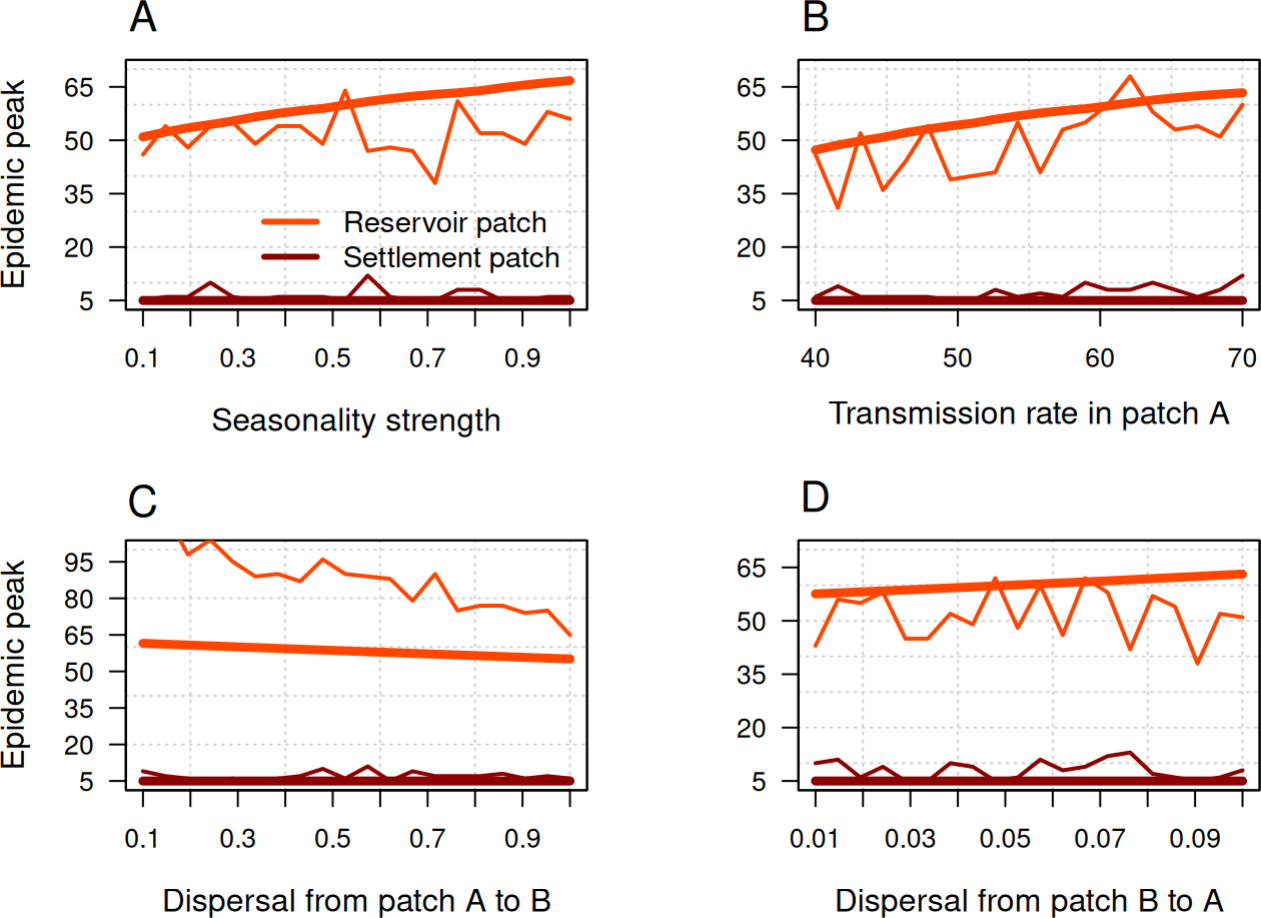
Seasonal disease transmission and seasonal human dispersal influence the Ebola epidemic peak. Changes in the epidemic peak as a function of the strength of seasonality (A), seasonal transmission rates in the reservoir patch (B), seasonal dispersal from the reservoir to the settlement (C), and seasonal dispersal from settlement to the reservoir patch (D) according to the deterministic (heavy line) and stochastic (thin line) models. Initial conditions for patch A were $S_{A}=95$, $I_{A}=5$ and $R_{A}=0$, and for patch B were $S_{B}=995$, $I_{B}=5$ and $R_{B}=0$. Other parameters were μ=0.2, γ=12, $\beta_{A}=60$, $\beta_{B}=5$, $\phi_{AB}=0.4$, $\phi_{BA}=0.04$ and σ=0.5.

**Figure 6.**
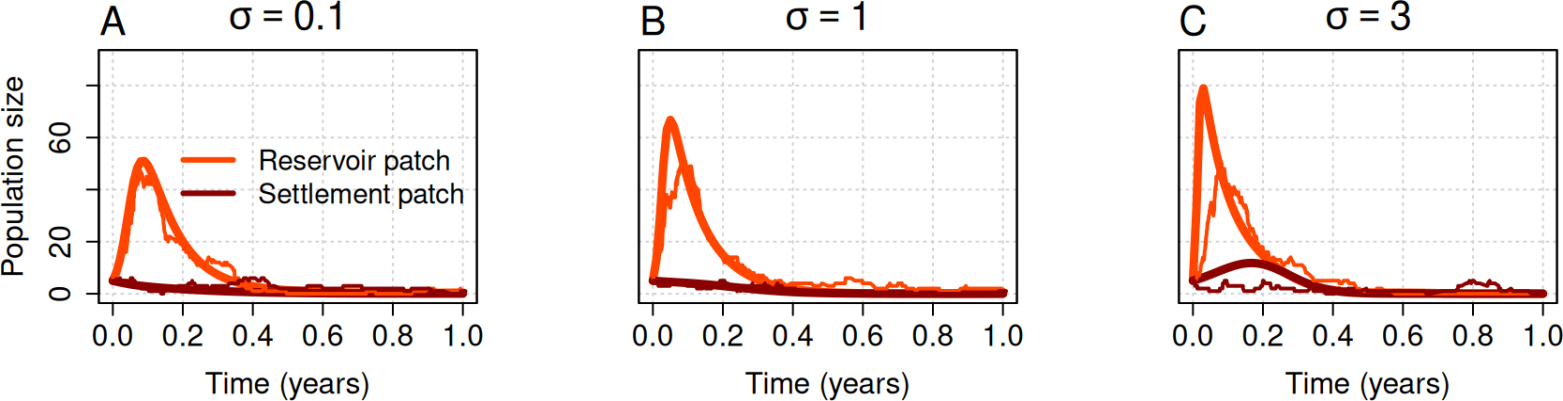
The epidemic peak increases with increasing strength of seasonality. The impact of seasonality on the epidemic peak in the deterministic (thick lines) and stochastic (thin lines) models for a period of one year. Initial conditions for patch A were $S_{A}=95$, $I_{A}=5$ and $R_{A}=0$, and for patch B were $S_{B}=995$, $I_{B}=5$ and $R_{B}=0$. Other parameters were μ=0.2, γ=12, $\beta_{A}=60$, $\beta_{B}=5$, $\phi_{AB}=0.4$, $\phi_{BA}=0.04$ and σ=0.5.

The amplitude of seasonality characterizes the strength of seasonal variations and their potential impact on the dynamics of the disease and has a direct influence on the peaks of seasonal transmission and movement rates. To understand this effect of amplitude, we evaluated the model over 1 year and investigated the effect of amplitude on the epidemic peak in both the reservoir and human settlement regions. The epidemic peak increases with the value of amplitude in both regions. Our results showed that the amplitude has a stronger influence in the reservoir region than in the human settlement region (figures 5A and 6). This strong influence on the reservoir region eventually impacts the settlement region as a result of human dispersal across the ecological boundary.

### 6.4. Oscillatory patterns and periodicity of infection clusters

**Figure 7.**
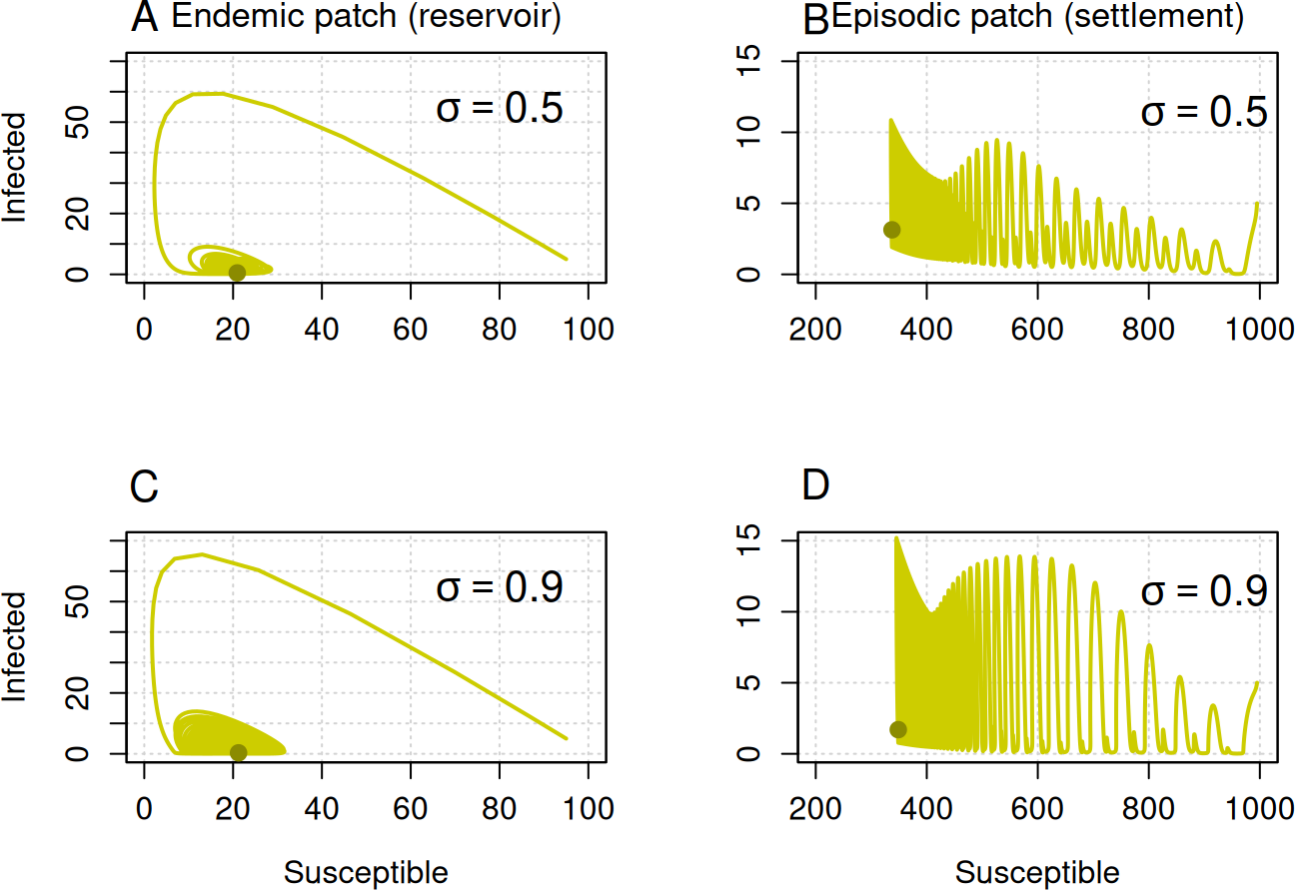
Seasonality amplitude alters the oscillatory patterns of the Ebola outbreak. Trajectories are displayed on a phase plane for two different values of the seasonality amplitude. The first column shows model trajectories in the reservoir patch, while the second column represents trajectories in the settlement patch. In all panels, the *x*-axis represents the number of susceptible individuals, and the *y*-axis represents the number of infected individuals. The green-filled circles indicate the endpoint of the trajectories. Initial conditions for patch A were $S_{A}=95$, $I_{A}=5$ and $R_{A}=0$, and for patch B were $S_{B}=995$, $I_{B}=5$ and $R_{B}=0$. Other parameters were μ=0.2, γ=12, $\beta_{A}=60$, $\beta_{B}=5$, $\phi_{AB}=0.4$, $\phi_{BA}=0.04$ and σ=0.5.

**Figure 8.**
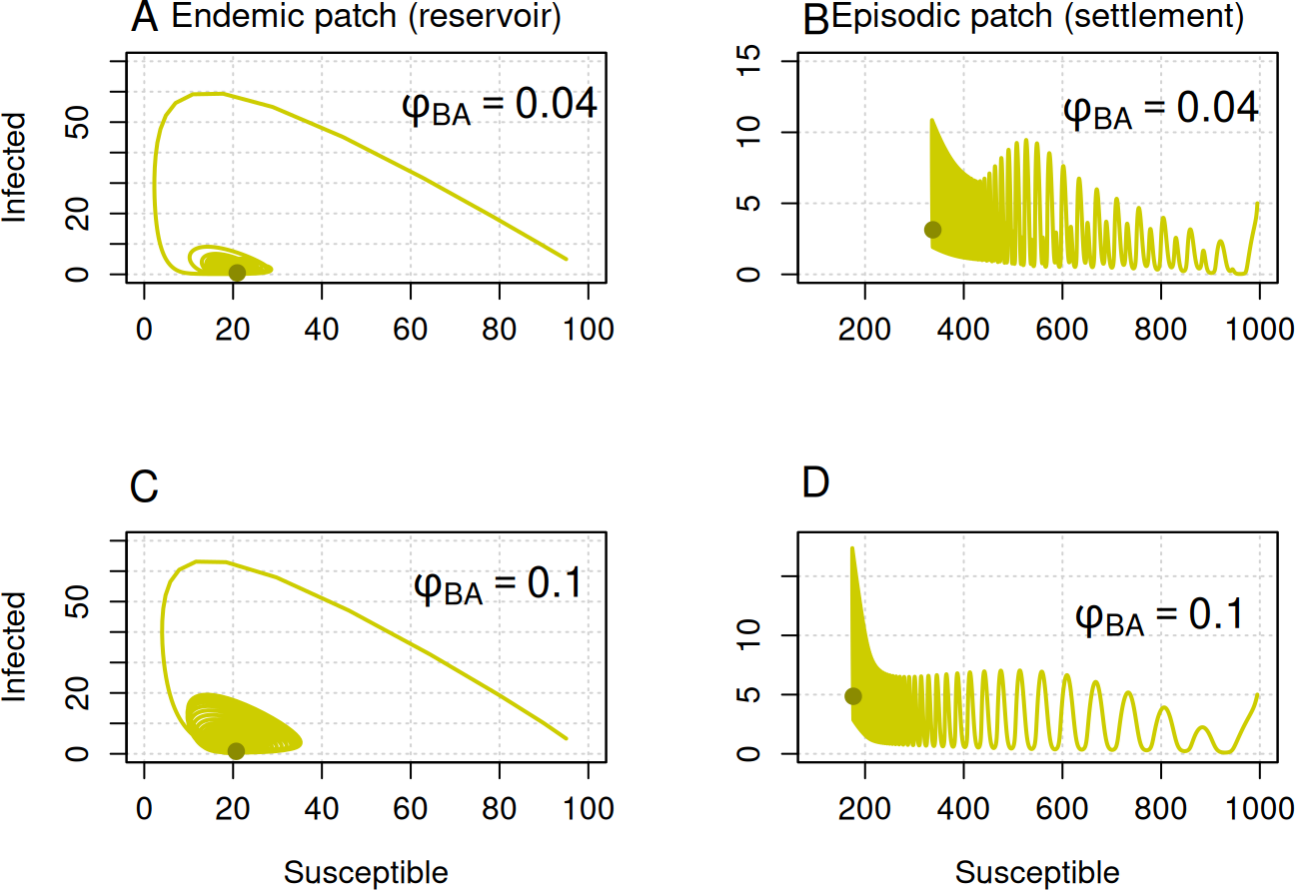
Dispersal magnitude alters the oscillatory patterns of the Ebola outbreak. Trajectories are displayed on a phase plane for two different values of human movement from settlement to reservoir patch. The first column shows model trajectories in the reservoir patch, while the second column represents trajectories in the settlement patch. In all panels, the *x*-axis represents the number of susceptible individuals, and the *y*-axis represents the number of infected individuals. The green circles indicate the endpoint of the trajectories. Initial conditions for patch A were $S_{A}=95$, $I_{A}=5$ and $R_{A}=0$, and for patch B were $S_{B}=995$, $I_{B}=5$ and $R_{B}=0$. Other parameters were μ=0.2, γ=12, $\beta_{A}=60$, $\beta_{B}=5$, $\phi_{AB}=0.4$, $\phi_{BA}=0.04$ and σ=0.5.

Solving the model for 100 years shows that damped oscillations in the number of infected individuals persist over time, as evidenced by the loops in the model trajectories (figure 7). In the reservoir patch, cyclic outbreaks occur with the periodicity of these oscillations increasing as the amplitude of seasonality rises from 0.5 to 0.9 (figure 8A,C). The settlement patch exhibits more frequent periodic oscillations, which become increasingly complex and sustained with higher seasonality amplitude (figure 7B,D). Increases in the amplitude of seasonality lead to increased dispersal to the reservoir patch and high transmissibility of the disease in both patches. These increases allow larger outbreaks in the human settlement region demonstrated by the higher number of infected individuals in our results (figure 7D). This suggests that increasing seasonality amplitude intensifies the periodicity of fluctuations, with a more pronounced effect seen in the human settlement patch. These findings suggest that seasonality may be responsible for the long-term persistence of periodic Ebola outbreaks in some regions.

However, stochastic results demonstrated that seasonal forcing does not significantly impact the periodicity of infection clusters (electronic supplementary material, appendix; figure A.3). The challenge in distinguishing between the effects of stochasticity and seasonality complicates the interpretation of seasonality’s role. Nevertheless, the stochastic model results suggest that increased amplitude of seasonality introduces greater variability in the timing of infection clusters, but does not substantially alter the overall pattern of periodic outbreaks (electronic supplementary material, appendix; figure A.3). As described above, fluctuation-driven extinction in the stochastic model significantly alters the patterns of periodicity predicted by the deterministic model. Our deterministic model demonstrates that higher seasonal amplitude drives the number of infected individuals close to the extinction boundary (figure 7), creating opportunities for stochasticity-driven extinctions of infection clusters. While the deterministic model suggests consistent periodic oscillations influenced by seasonality, the stochastic model shows that random fluctuations can lead to extinction events, disrupting these predicted patterns. This distinction highlights the potential limitations of deterministic models in accurately capturing the dynamics of infection clusters, particularly when stochastic effects are strong.

We varied the dispersal magnitude from 0.04 to 0.1 to investigate how human movement alters the disease trajectories and affects the stability of periodic fluctuations. Our results demonstrate that the magnitude of human dispersal from the settlement to the reservoir patch has significant effects on the oscillatory patterns, which are more pronounced in the settlement patch (figure 8). The amplitude of fluctuations is particularly decreased as the dispersal magnitude increases, leading to more stable periodic cycles of the disease in the human settlement patch (figure 8B,D). Increases in the dispersal magnitude cause a large shift in the disease trajectories on the phase plane, thereby reducing the number of susceptible individuals in the human settlement region. This suggests that dispersal increases spillover risk while promoting long-term stability of the period cycles.

## 7. Discussion

Outbreaks of zoonotic diseases, including a major epidemic of Ebola from 2014 to 2016, often have their origins in wildlife [77]. Because wildlife and humans typically occupy distinct local areas, the ecological boundary between human and animal populations is crucial for understanding the conditions that give rise to spillover [8]. It has long been suspected that Ebola spillover from wildlife into humans is linked to seasonal variations in climate conditions and human activities [46], perhaps due to temporal and spatial fluctuations in the abundance of resources. Food resources can be highly abundant in particular seasons of the year, attracting humans to reservoir regions where resources are found. We studied the influence of these seasonal forces on the dynamics and persistence of zoonotic diseases in human populations. We found that the impact of seasonality on the peak of the Ebola epidemic depends on the amplitude of these variations. These results suggest that large increases in resources in specific reservoir regions could potentially lead to an increased spillover risk.

Previous models developed to describe emerging spillover events from reservoirs into humans either neglected seasonality altogether [32] or considered the impact of seasonality on the spillover probability within a single-patch system [78,79]. These single-patch models account for seasonal changes by incorporating fluctuating disease transmission and recovery rates. It has been suggested that the influence of seasonality on spillover depends on several factors, particularly the time when an infectious individual is introduced and how seasonality is incorporated into infectious disease models. A multi-patch model of spillover also considered seasonality in both transmission and human dispersal rates [21]. That model aimed at understanding how spillover probability is influenced by the time and location at which an infectious individual is introduced to specific patches of the system. The findings indicated that seasonal transmission and dispersal patterns determine the times and patches associated with the highest outbreak risks. Our model was based on this framework, but we focused on different questions related to the drivers of the Ebola spillover at ecological boundaries.

In our study, we modelled a system comprising a small reservoir patch and a larger human settlement patch. Without human movement between the patches, outbreaks were confined to the reservoir. When dispersal was introduced, we assumed that individuals in the settlement patch did not interact directly with pathogens in the reservoir but instead transmitted infection via contact with other hosts in their own patch. Our results indicate that seasonal movement between the reservoir and settlement enabled Ebola spillover into the human population. Once introduced, the outbreak became established in the settlement and persisted. Spillover risk was primarily driven by two factors: the influx of susceptible individuals into the reservoir and the emigration of infected individuals from it. Ultimately, the disease faded from the system once extinction occurred in the reservoir source.

This model was developed as a conceptual framework to explore general features of zoonotic spillover at ecological boundaries, rather than to produce specific, operational forecasts. As such, it is intended for strategic understanding, not tactical prediction. One important limitation is that the model is not parameterized for any particular host–pathogen system, landscape or epidemiological setting. Although we have used parameter values informed by Ebola virus ecology, the results are illustrative rather than empirical. Furthermore, several key biological parameters, especially the amplitude of seasonal forcing in transmission and movement, remain poorly characterized in real-world systems, limiting our ability to evaluate model fit or realism. An additional simplification is that we do not distinguish host species. In situations where there would be a substantial number of animal hosts in the settlement patch or humans frequently visiting the reservoir patch, this structural assumption is expected to break down. Finally, we do not explicitly incorporate spatial heterogeneity, behavioural adaptations or policy interventions, which are important components of real spillover scenarios. Further development of the ideas developed here would benefit from integration with empirical data, especially concerning seasonal variation and patterns of human–wildlife interaction.

In summary, we conducted deterministic and stochastic analysis of a two-patch model to examine the effects of human movement and seasonal variation on spillover at ecological boundaries. Our results demonstrate that seasonal increases in transmission and dispersal rates can trigger spillover events that would not otherwise occur. These findings emphasize the importance of the settlement–wildland interface as a critical zone for the emergence of zoonotic diseases.

## Data Availability

No original data were collected in association with this study. R code to recreate the figures in this paper are available online [80]. Supplementary material is available online [81].
